# Tannic Acid-Copper Coordination Gel-Coated Mesoporous Cuprous Oxide Nanoplatform for Synergistic 5-FU Chemotherapy and Enhanced Chemodynamic Therapy

**DOI:** 10.3390/gels12060487

**Published:** 2026-06-02

**Authors:** Wenyao Zhang, Changjin Xu, Jiuyang Wang, Riqing Cheng, Huiqing Guo

**Affiliations:** College of Pharmacy, Inner Mongolia Medical University, Hohhot 010110, Chinarqcheng@immu.edu.cn (R.C.)

**Keywords:** TA, drug delivery, drug delivery system, CDT, synergistic therapy

## Abstract

To address the limitations of the tumor microenvironment (TME) and the inadequate efficacy of standalone chemodynamic therapy (CDT), this study developed a tannic acid-copper coordination gel-coated mesoporous Cu_2_O nanodelivery system (Cu_2_O@TA@5-FU) for synergistic enhanced CDT and chemotherapy. The system exhibits a high specific surface area (98 m^2^·g^−1^) and mesoporosity, achieving a 5-fluorouracil (5-FU) loading efficiency of 46.2%. Under simulated TME conditions, the nanodelivery system displayed markedly accelerated drug release and enhanced catalytic activity, indicative of pronounced TME responsiveness. In vitro, the Cu_2_O@TA support efficiently catalyzed a Fenton-like reaction with H_2_O_2_ to generate cytotoxic hydroxyl radicals (·OH) while depleting overexpressed intracellular GSH, thereby disrupting antioxidant defenses and amplifying oxidative stress. Combined with the antiproliferative action of released 5-FU, the synergistic treatment reduced 4T1 cell viability to approximately 23%, accompanied by sharp declines in intracellular ATP and GSH levels. This work overcomes the systemic toxicity of free 5-FU and the instability of Cu_2_O by employing a protective and stimuli-responsive TA-Cu coordination gel shell, offering a reliable strategy for TME-responsive synergistic nanotherapeutics that disrupt tumor metabolic and redox homeostasis.

## 1. Introduction

Malignant tumors represent a significant global public health challenge, and conventional clinical therapies are frequently constrained by limited efficacy and considerable toxicity, thereby necessitating the development of highly selective and low-toxicity treatment modalities [1]. The tumor microenvironment (TME) constitutes a complex niche essential for tumor cell survival and progression, characterized by distinct biochemical features absent in normal tissues, including mild acidity, elevated intracellular glutathione (GSH), and overexpressed hydrogen peroxide (H_2_O_2_) [2,3]. Stimuli-responsive nanomedicine delivery systems engineered to exploit TME characteristics facilitate tumor cell internalization and precisely controlled drug release with in situ activation of therapeutic functions under specific TME cues, a strategy that enhances antitumor outcomes while mitigating systemic toxicity [4].

Among emerging therapeutic approaches, chemodynamic therapy (CDT) utilizing cuprous oxide (Cu_2_O) nanomaterials has garnered substantial interest owing to the unique redox behavior and pronounced enzyme-mimetic catalytic activity of these materials [5,6,7]. Relative to traditional iron-based Fenton catalysts, Cu_2_O-mediated Fenton-like reactions operate across a broader pH range and sustain high catalytic efficiency within the mildly acidic TME [8,9]. Within this milieu, Cu_2_O degrades to release Cu^+^ ions that catalyze the conversion of endogenous H_2_O_2_ into highly cytotoxic hydroxyl radicals (·OH), thereby provoking oxidative injury and apoptosis. Concurrently, the liberated copper ions deplete overexpressed intracellular GSH while being reduced back to Cu^+^, thereby perpetuating a cyclic catalytic process [10]. This dual functionality of in situ reactive oxygen species (ROS) generation and antioxidant defense disruption substantially amplifies tumor oxidative stress [3,11,12,13]. Nevertheless, bare Cu_2_O nanoparticles are prone to rapid oxidation under physiological conditions, a process that compromises structural integrity and severely attenuates catalytic performance, thereby hindering in vivo translation [6,14,15].

The introduction of appropriate surface modifications is therefore essential. Gel-like interfacial coatings assembled via coordination chemistry, particularly metal-phenolic networks (MPNs), have emerged as promising constructs for intelligent nanomedicine platforms due to their dynamic crosslinking and stimuli-responsive properties [16,17,18]. Tannic acid (TA), a naturally abundant plant polyphenol, offers an attractive avenue for Cu_2_O stabilization and functionalization. TA exhibits favorable biocompatibility and biodegradability and possesses a molecular architecture enriched with pyrogallol and catechol moieties [18]. These functional groups confer several advantages. First, robust coordination between TA and copper ions facilitates rapid supramolecular self-assembly into a dense MPN gel shell that encapsulates the Cu_2_O surface, effectively shielding the core from ambient oxygen and preventing premature Cu^+^ oxidation and leakage [19]. Second, this three-dimensional gel coating enhances colloidal stability during circulation while supplying ample hydrogen-bonding and hydrophobic domains conducive to small-molecule chemotherapeutic loading. Third, the pH-labile nature of the coordination network ensures stability at physiological pH while enabling dissociation within the acidic TME, thereby achieving TME-responsive payload release [20,21]. The MPN gel layer thus serves concurrently as a biomimetic protective barrier, a robust platform for high-capacity drug loading via hydrophobic and hydrogen-bonding interactions, and a gatekeeper for pH-responsive controlled release.

Despite notable advances in CDT platforms, monotherapy is frequently hampered by insufficient intratumoral substrate availability, such as limited H_2_O_2_ levels, which impede complete tumor eradication [22,23]. The integration of CDT with conventional chemotherapy offers a rational strategy to surmount this constraint. 5-FU is a widely prescribed antimetabolite chemotherapeutic agent whose clinical utility is compromised by a short plasma half-life, poor tumor selectivity, low bioavailability, and propensity to elicit systemic toxicities including gastrointestinal distress and myelosuppression. Encapsulation of 5-FU not only circumvents its rapid systemic clearance but also leverages its mechanism of inhibiting thymidylate synthase, which impairs DNA repair and cell survival, thereby highly sensitizing tumor cells to CDT-induced oxidative stress [24,25]. The porous and hydrated nature of the TA-Cu coordination gel coating further facilitates high-capacity drug loading through hydrogen bonding and hydrophobic interactions. Overall, each component in this system plays a precise role: the Cu_2_O core serves as the nanozyme engine for CDT to generate ·OH; the TA network acts as a protective shield against premature oxidation, a pH-responsive gatekeeper, and a high-capacity sponge for drug loading; and 5-FU acts as the chemotherapeutic agent to halt cell cycle progression, working synergistically with CDT.

Therefore, to address the oxidative inactivation of Cu_2_O and the clinical limitations of free 5-FU, a novel nanocomposite (Cu_2_O@TA@5-FU) was designed and fabricated. This system utilizes a TA-coordinated MPN gel coating as a protective barrier to maintain Cu^+^ catalytic activity while facilitating efficient 5-FU loading. Upon exposure to the mildly acidic and GSH-rich TME, the gel shell undergoes responsive dissociation, triggering on-demand 5-FU release and in situ activation of the Cu_2_O core. The activated core amplifies oxidative stress via GSH depletion and ·OH generation, whereas the released 5-FU directly induces tumor cell apoptosis, achieving a highly synergistic antitumor outcome. By integrating an MPN coating with a Cu_2_O-based CDT system, the in vitro antitumor efficacy and underlying molecular mechanisms of this nanocomposite are systematically investigated, thereby providing a robust experimental foundation and theoretical framework for the further development of TME-responsive synergistic nanomedicines.

## 2. Results and Discussion

### 2.1. Structural and Surface Properties of the Prepared Cu_2_O@TA Material

**Figure 1:**
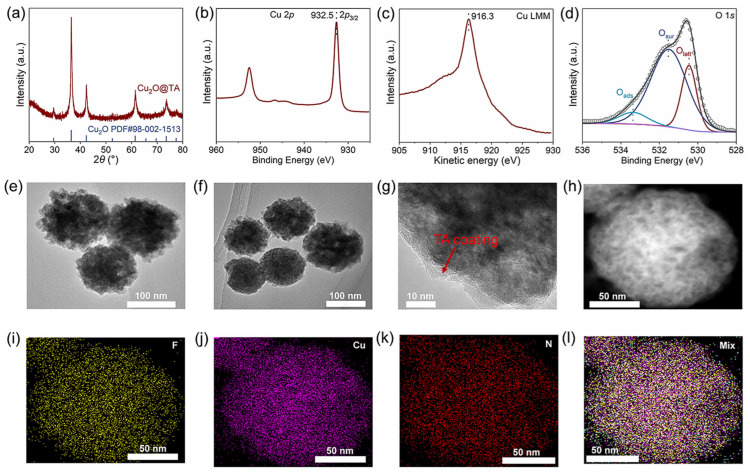
Structural and surface properties characterization of the as-prepared materials. (**a**) XRD pattern of the Cu_2_O@TA material; XPS spectra of Cu 2p (**b**), Cu LMM (**c**), and O 1s (**d**) of the Cu_2_O@TA material; TEM images of the Cu_2_O@TA (**e**) and Cu_2_O@TA@5-FU (**f**); (**g**) HRTEM image of the Cu_2_O@TA@5-FU; (**h**–**l**) HAADF-STEM image and corresponding mapping images of Cu_2_O@TA@5-FU. O 1s XPS: Circles: Raw experimental data; Black line: Overall fitted curve; Lowest pink line: Background; Other three colored lines: Deconvoluted peaks representing the three different oxygen species.

To fully elucidate the crystallographic structure, morphological features, and surface chemical states of the synthesized material, comprehensive characterizations were performed via XRD, TEM, and XPS. As shown in Figure 1a, the diffraction peak positions of Cu_2_O in the XRD pattern are in complete agreement with the standard card for Cu_2_O, confirming the successful synthesis of the Cu_2_O species. XPS analysis was further conducted to examine the surface chemical states. The Cu 2p spectrum (Figure 1b) exhibits a characteristic peak at a binding energy of 932.5 eV, which is attributable to reduced copper species (Cu^0^ or Cu^+^) [26,27]. Moreover, the absence of prominent Cu^2+^ satellite peaks in the 941–945 eV region suggests that copper species on the material surface exist predominantly in a reduced state [28]. Since the Cu 2p peak is insufficient to clearly discriminate between Cu^0^ and Cu^+^ [29], the Cu LMM Auger spectrum was further employed for identification. As presented in Figure 1c, the Auger spectrum displays a principal peak at a kinetic energy of 916.3 eV, which is assigned to Cu^+^ species [29], thereby verifying that the surface of Cu_2_O@TA is primarily composed of Cu^+^. The presence of Cu^+^ is favorable for mediating Fenton-like reactions and consequently enhancing antitumor performance. The high-resolution O 1 s spectrum (Figure 1d) was deconvoluted into three characteristic peaks. The peak located at 529.1 eV is assigned to lattice oxygen (O_latt_); the peak within the range of 530–534 eV corresponds to surface hydroxyl oxygen or carboxyl oxygen (O_sur_); and the peak near 534 eV is attributed to surface-adsorbed oxygen species (O_ads_) [30,31]. Notably, the O_ads_ are intimately linked to surface oxygen vacancies. The existence of these defective sites essentially facilitates the adsorption and subsequent activation of reactant molecules, thereby boosting the overall catalytic efficiency [32].

TEM observations were conducted to elucidate the morphological and elemental characteristics of the sample. As evidenced by Figure 1e, Cu_2_O@TA manifests as homogeneous spherical nanoparticles (~150 nm in diameter). The TEM image of the Cu_2_O@TA@5-FU sample (Figure 1f) further indicates that the spherical structure remains essentially unchanged following drug loading. The HRTEM image (Figure 1g) clearly shows a uniform organic coating layer on the exterior of the particles. Considering the coordination-driven self-assembly characteristics of tannic acid with copper ions, this coating can be identified as a MPN gel shell, confirming that tannic acid has been successfully encapsulated and a core–shell structure has been formed. Furthermore, HAADF-STEM imaging coupled with elemental mapping analysis (Figure 1h–l) demonstrates that N, F, and O elements are uniformly distributed within the nanoparticles. This observation verifies that 5-FU has been successfully loaded and homogeneously dispersed throughout the composite, thereby confirming the successful construction of the nanodrug delivery system. DLS analysis was further employed to evaluate the hydrodynamic size and colloidal stability of Cu_2_O@TA in PBS. As shown in Figure S2, the nanoparticles exhibit a monomodal size distribution with an average hydrodynamic diameter of 105.2 nm and a low polydispersity index (PDI) of 0.146, indicating excellent monodispersity and the absence of significant aggregation. The slight difference between the DLS-derived hydrodynamic size (105.2 nm) and the TEM-measured core size (~150 nm, Figure 1e) is attributed to the fact that DLS measures the hydrated particle diameter including the TA-Cu coordination gel shell, whereas TEM reveals the dry core after sample preparation. The low PDI value (<0.2) further supports the uniform size distribution observed in TEM images, confirming that the Cu_2_O@TA nanoparticles remain well-dispersed without aggregation under physiological conditions.

**Figure 2:**
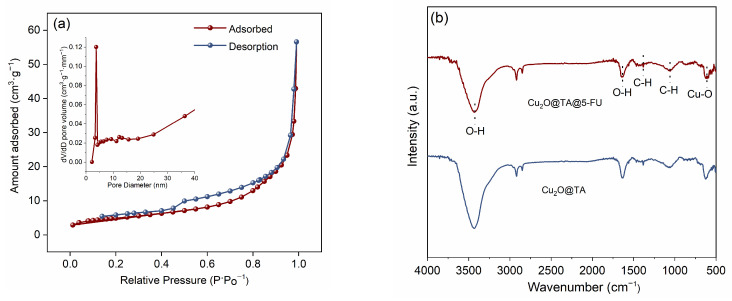
Textural properties of the prepared Cu_2_O@TA-based materials. (**a**) N_2_ adsorption–desorption isotherms of the Cu_2_O@TA material (Insert: pore size distributions); (**b**) FTIR spectra of the prepared Cu_2_O@TA and Cu_2_O@TA@5-FU materials.

To elucidate the textural properties of the synthesized material, N_2_ adsorption–desorption porosimetry was performed on the Cu_2_O@TA sample. As illustrated in Figure 2a, the composite displays a classic Type III isotherm featuring an H3 hysteresis loop within the relative pressure (P/P_0_) range of 0.4–1.0. According to the IUPAC classification, this profile is indicative of slit-like interstitial spaces, implying that the weak pore structures primarily originate from the interstitial packing of the nanoparticles [33]. The BET measurement reveals that Cu_2_O@TA possesses a specific surface area of 98 m^2^·g^−1^. While further analysis using the BJH model shows a minor pore size distribution peak at approximately 3 nm, quantitative analysis reveals that the overall porosity is highly limited. Specifically, the total pore volume is only 0.09 cm^3^/g, with the volume of pores smaller than 5 nm (the main peak) being merely 0.008 cm^3^/g. Considering these minimal pore volumes and the moderate specific surface area, the rigid physical pore structures alone are structurally insufficient to accommodate the substantial amount of 5-FU loaded into the nanocarrier. Therefore, rather than being strictly confined within rigid mesopores, it is evident that a significant portion of the drug molecules is effectively entrapped within the flexible TA-Cu coordination gel network, alongside physical adsorption on the support surface. This combined mechanism of surface adsorption and flexible gel network encapsulation fundamentally explains the high drug loading capacity achieved by this nanoplatform.

FTIR spectroscopy was employed to characterize the Cu_2_O@TA support and the drug-loaded Cu_2_O@TA@5-FU sample (Figure 2b). The results show that both samples exhibit a characteristic O–H stretching vibration peak near 3500 cm^−1^ and a characteristic absorption peak corresponding to the Cu–O bond in the 500–600 cm^−1^ region [34], indicating that the drug loading process did not disrupt the skeletal structure of Cu_2_O@TA. The drug-loaded sample displays characteristic C–H vibration absorption peaks in the 1000–1600 cm^−1^ range [35], and the overall spectral profile closely resembles that of the carrier, with no independent characteristic vibration peaks attributable to crystalline 5-FU being observed. These findings suggest that the loaded 5-FU drug does not exist as aggregated independent crystals, but rather is uniformly dispersed on the crystalline Cu_2_O@TA support surface in an amorphous or molecularly adsorbed state. This interpretation is consistent with the BET analysis showing that the high specific surface area of the carrier provides sufficient adsorption sites and with the TEM observations revealing visually well-dispersed particles without obvious large-scale aggregation in the dry state.

### 2.2. Drug Loading and Release Performance of Cu_2_O@TA@5-FU

**Figure 3:**
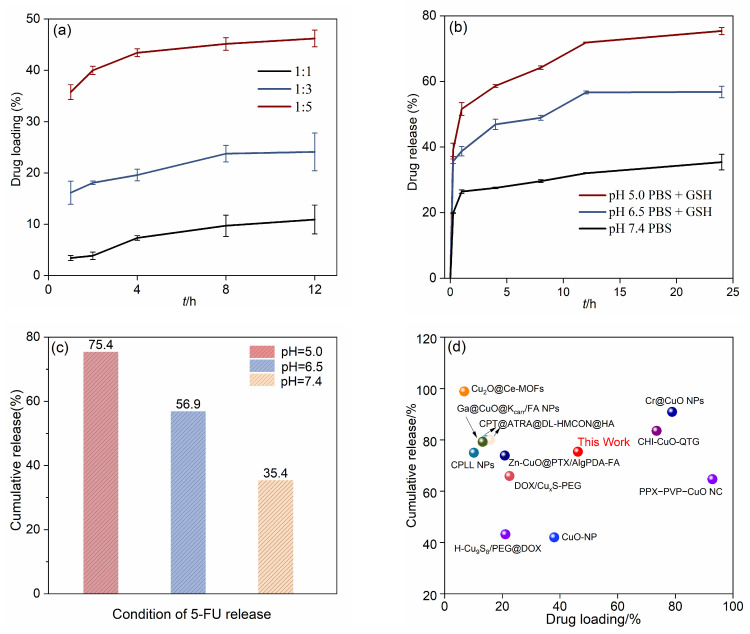
Drug performance of Cu_2_O@TA material. (**a**) Drug loading; (**b**) Drug release; (**c**) 5-FU cumulative release; (**d**) Comparison of drug loading and release performance.

To evaluate the drug loading performance of the Cu_2_O@TA material, the effects of the feed ratio (support-to-drug mass ratio) and loading duration on the drug loading capacity were systematically investigated (Figure 3a). The results indicate that the drug loading capacity exhibits a marked increasing trend as the feed ratio is gradually elevated from 1:1 to 1:5. Taking both drug loading efficiency and drug utilization into careful consideration, the optimal loading conditions were determined to be a feed ratio of 1:5 and a loading duration of 12 h. Under these conditions, the drug loading efficiency reached 46.2%, further confirming that the support, by virtue of its high specific surface area and abundant mesoporous architecture, possesses favorable drug adsorption and loading capability.

The responsive drug release behavior of the material was further evaluated by simulating the characteristics of the tumor microenvironment. The drug-loaded system Cu_2_O@TA@5-FU was separately placed in PBS with different pH values (7.4, 6.5, and 5.0) and in PBS solution containing reduced GSH, and the cumulative release profiles of 5-FU were monitored (Figure 3b). The drug delivery system exhibited pronounced pH/GSH dual-responsive release characteristics. Under pH 7.4 conditions simulating the normal physiological environment, the drug release was slow, and the cumulative release percentage over 24 h was relatively low, indicating favorable structural stability of the gel coating during blood circulation. In contrast, in the medium simulating the tumor microenvironment with acidic conditions (pH 5.0) containing GSH, the drug release rate was significantly accelerated, and the cumulative release percentage over 24 h reached 75.4% (Figure 3c). This responsive release behavior is primarily attributed to the protonation-induced disassembly of the MPN gel shell of Cu_2_O@TA in the acidic environment, while GSH promotes the reductive dissolution of Cu^+^, leading to the collapse of the support framework. Consequently, rapid and controllable drug release at the target site is achieved. Collectively, these results demonstrate that Cu_2_O@TA@5-FU can specifically respond to the weakly acidic conditions and elevated GSH levels characteristic of the tumor microenvironment, enabling intelligent and controllable drug delivery and exhibiting considerable promise for applications in tumor-targeted chemotherapy. To further elucidate the release mechanism, the in vitro release data at pH 5.0 with GSH were analyzed using various kinetic models. The release profile exhibited the best fit with the Korsmeyer-Peppas model (R^2^ = 0.9537), with a release exponent (0.17) indicating a pseudo-Fickian diffusion mechanism (Figure S1). This suggests that the rapid release of 5-FU is primarily governed by drug diffusion through the porous TA-Cu MPN coordination gel shell, which is facilitated by the protonation-induced loosening of the shell in the acidic environment. Furthermore, compared with previous studies [7,8,34,36,37,38,39,40,41,42,43], as shown in Figure 3d, the Cu_2_O@TA@5-FU platform exhibits a highly competitive drug-loading capacity alongside an excellent TME-triggered release profile, highlighting its structural superiority.

### 2.3. Cu_2_O@TA-Mediated OH Generation and GSH Depletion

**Figure 4:**
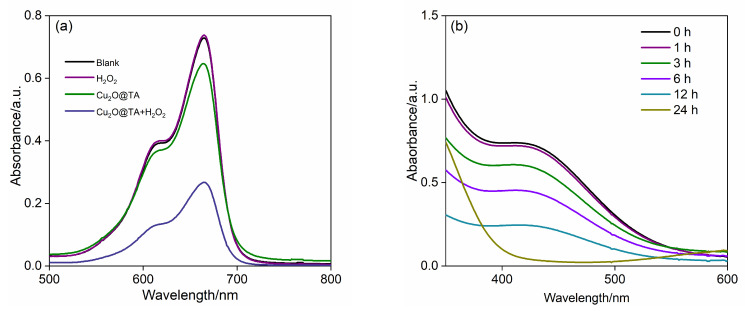
Evaluation of Cu_2_O@TA nanocomposites for enhancing chemodynamic therapy. (**a**) OH generation catalyzed by Cu_2_O@TA in the presence of H_2_O_2_, detected using MB as an oxidative probe; (**b**) Depletion of GSH after incubation with Cu_2_O@TA, measured via the DTNB assay.

To verify the catalytic performance of the Cu_2_O@TA nanomaterial for chemodynamic therapy, its capacity to generate ·OH in the presence of H_2_O_2_ was first evaluated. MB was employed as a ·OH indicator, and the attenuation of its characteristic absorption peak at 665 nm was monitored to indirectly reflect the level of ·OH production. As shown in Figure 4a, the absorbance of MB at 665 nm decreased substantially when both Cu_2_O@TA and H_2_O_2_ coexisted in the reaction system, indicating that Cu_2_O@TA can effectively catalyze a Fenton-like reaction with H_2_O_2_ to produce ·OH possessing strong oxidative activity. This result demonstrates that Cu_2_O@TA can mediate in situ ·OH generation under conditions simulating the tumor microenvironment, highlighting its potential application in chemodynamic therapy.

Given that elevated concentrations of GSH within tumor cells can scavenge ·OH and consequently diminish the efficacy of chemodynamic therapy, the construction of a nanosystem capable of concurrently depleting GSH is of considerable significance for enhancing therapeutic outcomes. The ability of Cu_2_O@TA to modulate GSH levels was further quantitatively analyzed using the Ellman’s reagent method. The underlying principle involves the specific reaction of DTNB with GSH to yield a yellow product, TNB2-, which exhibits characteristic absorption at approximately 412 nm. As presented in Figure 4b, the absorbance at 412 nm decreased in a time-dependent manner with prolonged coincubation of Cu_2_O@TA and GSH, signifying effective GSH elimination. Collectively, these findings demonstrate that the Cu_2_O@TA nanosystem can efficiently reduce intracellular GSH levels and disrupt the antioxidant defense system of tumor cells, thereby providing critical support for enhancing the efficacy of chemodynamic therapy.

### 2.4. In Vitro Cell Viability Assay

**Figure 5:**
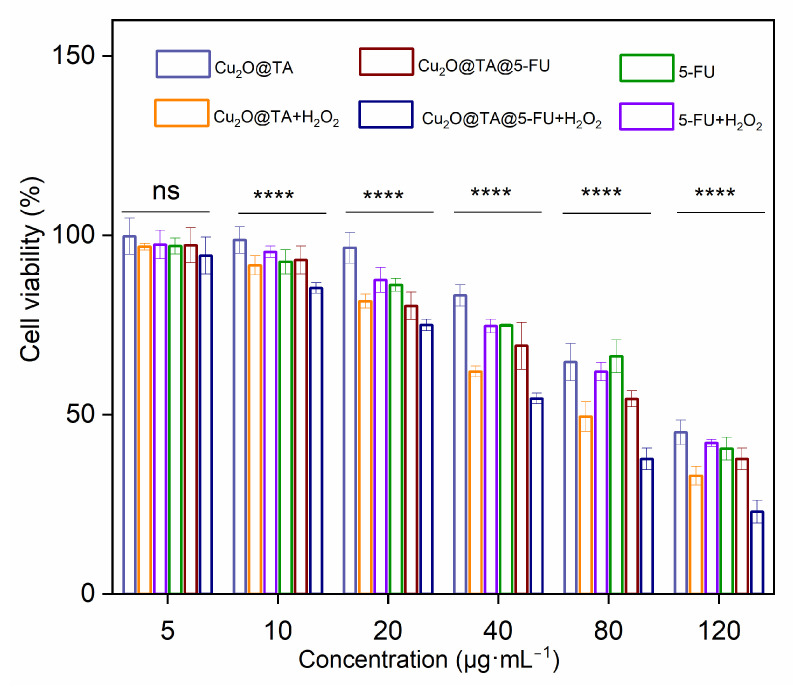
Survival rate of 4T1 cells treated with Cu_2_O/TA, H_2_O_2_ + Cu_2_O/TA, 5-FU, H_2_O_2_ + 5-FU, H_2_O_2_ + Cu_2_O/TA@5-FU and Cu_2_O/TA@5-FU. ns: no significant differences, **** *p* < 0.0001.

To validate the synergistic antitumor efficacy of the engineered nanoplatform, a CCK-8 assay was utilized to assess the in vitro cytotoxicity across various formulations against 4T1 cells under TME-mimicking conditions (Figure 5). The experimental results demonstrated pronounced concentration-dependent cytotoxicity across all treatment groups. Firstly, the addition of exogenous H_2_O_2_ alone did not significantly alter the cytotoxic effect of 5-FU, as evidenced by the lack of a significant difference in cell viability between the 5-FU group and the 5-FU + H_2_O_2_ group. This finding excludes any direct interference of H_2_O_2_ itself with cell proliferation and establishes the baseline cytotoxic effect of free 5-FU as a chemotherapeutic agent. In the assessment of the CDT effect, treatment with the unloaded Cu_2_O@TA material alone induced a certain degree of cell death, a phenomenon attributable in part to the Fenton-like reaction between the material and trace levels of endogenous H_2_O_2_ within the cells. Upon the introduction of additional H_2_O_2_ to mimic the elevated oxidative stress characteristic of the TME (the Cu_2_O@TA + H_2_O_2_ group), cell viability at equivalent concentrations decreased further and more significantly compared with the Cu_2_O@TA group alone. This result convincingly substantiates that the Cu^+^ species released from the material can efficiently catalyze a Fenton-like reaction with H_2_O_2_ to generate substantial quantities of ·OH, thereby exacerbating oxidative damage and apoptosis in tumor cells.

More importantly, the cytotoxic effect of the drug-loaded composite Cu_2_O@TA@5-FU in the absence of exogenous H_2_O_2_ was comparable to that of free 5-FU at the same concentration, indicating that the nanocarrier not only preserves the bioactivity of the drug but also exhibits favorable drug release performance. When exogenous H_2_O_2_ was introduced into the system, the combination therapy group (Cu_2_O@TA@5-FU + H_2_O_2_) displayed the most potent cytotoxicity. The cell-killing effect was further enhanced with increasing material concentration, and at the highest tested concentration (120 μg·mL^−1^), cell viability plummeted to approximately 23% (*p* < 0.0001). Although direct cellular uptake pathways were not visualized in this study, it is a well-established consensus in nanomedicine that nanoparticles with a size of approximately 150 nm are predominantly internalized into tumor cells via endocytosis [32]. Following this endocytic internalization, the nanoparticles are entrapped in acidic endo/lysosomes, where the low pH and highly reductive environment trigger the rapid disassembly of the TA coating. This leads to the endosomal escape and cytoplasmic release of 5-FU and Cu ions, thereby initiating their respective pharmacological cascades. The remarkable cytotoxicity of the combination group is not merely an additive effect but a true synergistic interaction. 5-FU induces cell cycle arrest and DNA damage, which compromise the cell’s ability to repair itself. Simultaneously, the CDT process floods the compromised cells with highly reactive ·OH radicals. This dual-pronged attack overwhelms the cellular repair machinery, leading to the observed severe cell death. In summary, the Cu_2_O@TA@5-FU nanoplatform achieves highly efficient eradication of 4T1 tumor cells under simulated TME conditions through the synergistic action of CDT and chemotherapy. These findings provide a robust in vitro experimental foundation for the application of Cu_2_O@TA-based nanodrug delivery systems in combined tumor-targeted therapy.

### 2.5. Evaluation of Cellular ATP and GSH Contents

In evaluating the interventional effects of nanomaterials on tumor cells, simultaneous detection of intracellular ATP and GSH levels provides critical reference values. ATP serves as a core indicator of cellular energy metabolism, and alterations in its level directly reflect the metabolic activity status of cells. GSH, as a pivotal intracellular antioxidant molecule, plays a central role in maintaining redox homeostasis, and its concentration is directly linked to the capacity of cells to scavenge free radicals and resist oxidative stress. Through the combined measurement of these two molecules, the regulatory effects of nanomaterials on tumor cells can be assessed from the dual perspectives of energy metabolism and oxidative defense, thereby furnishing key evidence for elucidating their antitumor mechanisms.

**Figure 6:**
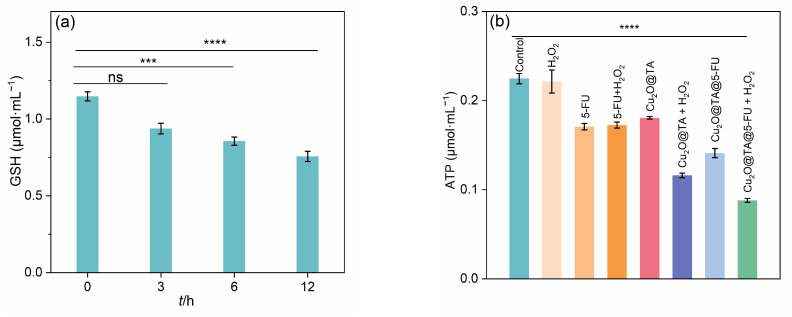
Effects of various treatments on intracellular GSH and adenosine triphosphate (ATP) levels in 4T1 cells. (**a**) Intracellular GSH levels after incubation with Cu_2_O@TA at different time points; (**b**) The ATP level of 4T1 cells treated with Cu_2_O@TA, H_2_O_2_ + 5-FU, H_2_O_2_ + Cu_2_O@TA@5-FU, H_2_O_2_ + Cu_2_O@TA, Cu_2_O@TA@5-FU and 5-FU. n = 3, mean ± SD. ns: no significant differences, *** *p* < 0.001, **** *p* < 0.0001.

As shown in Figure 6a, the intracellular GSH concentration exhibits a gradual declining trend over time following treatment with Cu_2_O@TA, indicating that the material effectively depletes intracellular GSH and compromises the antioxidant defense capacity of tumor cells. This process may further exacerbate cytotoxicity mediated by oxidative stress, suggesting a potential role of the material in promoting tumor cell apoptosis. Variations in intracellular ATP levels in 4T1 cells under different treatment conditions are presented in Figure 6b. Compared with the control group, treatment with H_2_O_2_ alone did not induce a significant change in ATP levels, indicating that exogenous H_2_O_2_ itself exerts no appreciable interference with basal cellular energy metabolism. Treatment with either Cu_2_O@TA alone or 5-FU alone led to a comparable degree of reduction in ATP content. The Cu_2_O@TA@5-FU group without exogenous H_2_O_2_ exhibited a more pronounced inhibitory effect on ATP content, indicating that the drug-loaded system can disrupt cellular energy metabolism through synergistic mechanisms even in the absence of exogenous H_2_O_2_. Notably, in the presence of H_2_O_2_, Cu_2_O@TA significantly reduced intracellular ATP content, whereas the Cu_2_O@TA@5-FU drug delivery system exerted an even more prominent suppressive effect on ATP under identical conditions. The profound depletion of intracellular GSH cripples the tumor cells’ intrinsic antioxidant defenses, rendering them highly susceptible to ROS-mediated damage. The burst of ·OH radicals generated via the Cu-based Fenton-like reaction directly attacks cellular macromolecules, particularly inducing mitochondrial dysfunction. Since mitochondria are the primary sites of ATP synthesis, such severe oxidative stress leads to a sharp decline in ATP levels, subsequently triggering apoptotic cascades [44,45]. Furthermore, the incorporation of 5-FU synergizes with this process; while 5-FU directly impairs DNA replication, the CDT-induced ROS exacerbates cellular stress, creating a lethal cycle of redox imbalance and metabolic collapse [46]. Collectively, these results demonstrate that under simulated tumor microenvironment conditions, the nanomaterial can effectively perturb the energy metabolism of tumor cells by catalyzing the production of reactive oxygen species (ROS), thereby suppressing their metabolic activity. This finding corroborates the aforementioned cytotoxicity assay results and further substantiates the conclusion that Cu_2_O@TA@5-FU achieves highly efficient antitumor efficacy through the synergistic interplay of chemodynamic therapy and chemotherapy.

### 2.6. Study Limitations

While this study establishes a robust in vitro proof-of-concept for the Cu_2_O@TA@5-FU nanoplatform, certain aspects warrant further investigation for preclinical translation. Although current assays confirmed TME-responsive ROS generation and GSH depletion, future studies incorporating specific pharmacological modulators (e.g., NAC or BSO) will help precisely benchmark these catalytic mechanisms. Furthermore, transitioning this smart delivery system toward in vivo applications will require comprehensive validations, including pharmacokinetics, biodistribution, and biosafety in tumor xenograft models, alongside therapeutic selectivity evaluations on normal mammalian cell lines.

## 3. Conclusions

In summary, this study successfully developed a dual TME-responsive Cu_2_O@TA@5-FU nanodrug delivery system for synergistic enhanced CDT and chemotherapy. An MPN protective gel layer was constructed on mesoporous Cu_2_O via coordination-driven self-assembly of TA and Cu^+^, forming a stable and responsive gel coating. This design prevents premature oxidation of the Cu_2_O core to preserve catalytic activity while providing ample drug loading capacity (46.2%). Under the mildly acidic, high-GSH TME conditions, the nanosystem intelligently disassembles via gel shell degradation, enabling targeted 5-FU release and in situ Cu^+^ generation. Mechanistic studies revealed that released Cu^+^ significantly depletes intracellular GSH and catalyzes the conversion of endogenous H_2_O_2_ into ·OH radicals. Subjected to this dual CDT/chemotherapy assault, 4T1 tumor cells exhibited markedly suppressed ATP and GSH levels, with cell viability falling to 23%. This work offers a novel strategy to mitigate the premature oxidation of copper-based nanozymes and establishes an in vitro foundation for designing responsive synergistic nanoplatforms for cancer therapy. Future in vivo studies will be essential to validate its clinical translational potential.

## 4. Materials and Methods

### 4.1. Materials

Copper(II) chloride dihydrate (CuCl_2_·2H_2_O) and sodium hydroxide (NaOH) were obtained from Xilong Scientific Co., Ltd. (Guangzhou, China). Tannic acid (TA) and ascorbic acid were purchased from Shanghai Aladdin Biochemical Technology Co., Ltd. (Guangzhou, China). Phosphate-buffered saline (PBS, pH 5.0, 6.5, and 7.4) and 5-fluorouracil (5-FU) were acquired from Shanghai Yuanye Bio-Technology Co., Ltd. (Shanghai, China). Methanol was supplied by Thermo Fisher Scientific (Scoresby, VIC, Australia). Methylene blue (MB) and 5,5′-dithiobis(2-nitrobenzoic acid) (DTNB) were sourced from Shandong Keyuan Biochemical Co., Ltd. (Laizhou, China). Hydrogen peroxide (H_2_O_2_) was obtained from Shanghai Macklin Biochemical Co., Ltd. (Shanghai, China). Glutathione (GSH) was procured from Beijing Coolaber Technology Co., Ltd. (Beijing, China). All materials were applied in their original form with no additional purification procedures.

### 4.2. Synthesis of Spherical Cu_2_O@TA

Initially, an aqueous copper solution was formulated using 1.704 g of CuCl_2_·2H_2_O and 80 mL of deionized water. The Cu(OH)_2_ precursor was subsequently precipitated by adding 20 mL of a 2 M NaOH solution. Finally, the suspended product was separated by centrifugation, thoroughly washed with pure water, and dispersed again into 100 mL of H_2_O. A tannic acid solution (1 mL, 40 mg·mL^−1^) was then slowly added dropwise, and the mixture was stirred for an additional 30 min to enable the coordination-driven coating of TA onto the Cu(OH)_2_ precursor surface, forming a self-assembled TA-Cu coordination gel layer. The Cu(OH)_2_@TA complex was recovered by centrifugation and washed to remove unbound TA.

The as-prepared Cu(OH)_2_@TA precipitate was dispersed into 80 mL of purified water. Subsequently, to trigger the reduction process, 20 mL of a 0.25 M ascorbic acid solution was slowly dripped into the suspension while maintaining continuous stirring. The reaction was allowed to proceed for 30 min to achieve the in situ reduction of Cu(OH)_2_ to Cu_2_O while preserving the TA gel coating. The resulting product was washed repeatedly with deionized water by centrifugation to eliminate residual reactants and subsequently lyophilized for 24 h to yield the purified Cu_2_O@TA nanocomposite. Based on the EDS elemental analysis (Table S1), the carbon content is approximately 4.59 wt%. Considering the theoretical carbon mass fraction in the molecular formula of tannic acid, the TA content in the Cu_2_O@TA carrier is estimated to be about 8.56 wt%.

### 4.3. Preparation of Cu_2_O@TA@5-FU Drug-Loaded System

A 5-FU stock solution (10 mg·mL^−1^) was prepared by dissolving a precisely weighed amount of 5-FU in a methanol–water mixed solvent (10/90 volume ratio) with ultrasonication. The Cu_2_O@TA nanocomposite was then uniformly dispersed in the 5-FU solution at a predetermined mass ratio. The mixture was placed in a thermostatic shaker and agitated under controlled speed and temperature for a specified duration to ensure sufficient drug adsorption and loading onto the carrier. Following the loading period, the solid product was collected by centrifugation, washed with deionized water to remove any surface-bound free drug, and the final Cu_2_O@TA@5-FU drug-loaded system was obtained.

### 4.4. Material Characterization

The crystal structure of the synthesized Cu_2_O@TA nanomaterial was characterized by powder X-ray diffraction (XRD) to confirm its phase composition. The morphology, size distribution, and surface structural features were observed using transmission electron microscopy (TEM). The pore structure was systematically characterized via nitrogen adsorption–desorption techniques, employing the Brunauer–Emmett–Teller (BET) and Barrett-Joyner-Halenda (BJH) models to quantify the specific surface area and pore size distribution. In terms of surface chemistry, Fourier transform infrared (FTIR) spectroscopy was carried out. The recorded characteristic absorption bands allowed for the precise determination of molecular bonds and surface functional groups, offering detailed insights into the structural makeup.

### 4.5. Drug Loading Performance Test of Cu_2_O@TA Material

5-FU was loaded onto the surface of the Cu_2_O@TA material via an impregnation method. The specific procedure was as follows. Precisely weighed 5-FU was dissolved in 10 mL of mobile phase under continuous stirring until complete dissolution. A designated amount of the Cu_2_O@TA composite was subsequently added to the solution and uniformly dispersed through ultrasonic treatment. Adsorption kinetic profiles were established by periodically extracting 1 mL samples from the suspension. After removing the solid particles via a 0.22 μm membrane filter, the remaining 5-FU concentration in the filtered solution was analyzed via high-performance liquid chromatography (HPLC) at a measurement wavelength of 265 nm. Upon completion of the adsorption process, the residual mixture was subjected to centrifugation, and the resulting solid precipitate was collected and vacuum dried to obtain the 5-FU-loaded Cu_2_O@TA drug delivery system. The loading capacity of the carrier for 5-FU was quantitatively evaluated by calculating the drug loading efficiency (DL) according to Equation (1).

(1) $$DL\left(\%\right)=\frac{W-W_{0}}{W_{1}}\times 100\%$$

where *W* is the total initial mass of 5-FU added, *W*_0_ represents the mass of 5-FU remaining in the supernatant, and *W*_1_ denotes the total mass of the final carrier together with the loaded drug.

### 4.6. In Vitro Drug Release Performance of Cu_2_O@TA@5-FU Material

The dynamic dialysis method was employed to investigate the drug release behavior of the drug-loaded material under different pH conditions. An appropriate amount of the drug-loaded material was sealed inside a dialysis bag with a molecular weight cutoff of 5000 Da, and a release medium of the corresponding pH value was added. The dialysis bag was subsequently immersed in a container holding 12 mL of phosphate-buffered saline (0.01 mol/L, pH values of 7.4, 6.5, and 5.0, respectively) to establish the in vitro release system. The entire system was placed in a thermostatic shaker and incubated continuously at 37 °C with a shaking speed of 100 r·min^−1^ to simulate the physiological environment in vivo. To monitor the in vitro release profile, 1 mL samples were periodically removed from the system and rapidly replaced with 1 mL of fresh PBS (adjusted to the corresponding temperature and pH). The extracted fractions were clarified using a 0.22 μm pore size filter before determining the 5-FU concentration via HPLC at 265 nm. Ultimately, Equation (2) was applied to calculate the cumulative percentage of the released drug, facilitating the kinetic evaluation of the drug-loaded matrix under various pH conditions.

(2) $$E(\%)=\frac{C_{n}\times V+\sum_{1}^{n-1}C_{k}\times V_{0}}{m}\times 100\%$$

where *E* denotes the cumulative drug release percentage; *C_n_* represents the mass concentration of 5-FU in the release medium at the n-th sampling interval (μg·mL^−1^); *V* is the total volume of the release medium; *C*_k_ represents the mass concentration of the drug in the release medium at the k-th sampling interval; *V*_0_ denotes the volume withdrawn at each sampling interval; and n indicates the number of sampling intervals.

### 4.7. Detection of ROS Generation Ability of Cu_2_O@TA Material

MB was employed as a specific probe to detect the production of·OH in the system. An MB solution containing 5 mmol·L^−1^ H_2_O_2_ was added to phosphate-buffered saline (pH 5.0) containing Cu_2_O@TA at a concentration of 120 μg·mL^−1^ to achieve a final MB concentration of 0.15 mmol·L^−1^. After thorough mixing, the system was incubated at 37 °C for 30 min. Following incubation, the absorbance of the reaction mixture was measured at a wavelength of 665 nm using an ultraviolet-visible spectrophotometer to characterize the Fenton-like catalytic activity of the material.

### 4.8. Detection of GSH Consumption Ability of Cu_2_O@TA Material

To further investigate the in vitro glutathione depletion capacity of the material, Cu_2_O@TA at various concentrations was mixed with GSH solution and incubated for different durations of 0, 3, 6, and 12 h, respectively. Following incubation, DTNB was utilized as a probe for the quantitative detection of residual GSH in the system. The specific interaction between DTNB and the sulfhydryl moiety of GSH yields a yellow complex, providing a basis for quantification. To evaluate the GSH consumption efficiency of Cu_2_O@TA, the characteristic absorbance of this colored solution was monitored at 412 nm using an ultraviolet-visible (UV-Vis) spectrophotometer.

### 4.9. Intracellular ATP and GSH Assay

To evaluate the intracellular concentrations of ATP and GSH, 4T1 cells were plated into 12-well plates at 2 × 10^5^ cells/well and incubated for 12 h to allow for cell attachment. Following this adhesion phase, the cells were exposed to the designated formulations at varying concentrations for an additional 24 h. After treatment, the cells were collected, lysed with the corresponding lysis buffer, and centrifuged. The resulting supernatant was used to quantify ATP and GSH levels employing an ATP assay kit (Beyotime, Shanghai, China) and Ellman’s reagent (Solarbio, Beijing, China), respectively. Protein concentrations were determined by the BCA method for normalization of the results.

### 4.10. In Vitro Cytotoxicity Assay

The standard Cell Counting Kit-8 (CCK-8) protocol was employed to investigate the in vitro cytotoxicity of the Cu_2_O@TA-based formulations. Initially, 4T1 cells were plated in 96-well plates and incubated for 12 h to facilitate cell adherence. Following the aspiration of the spent medium, the cells were exposed to fresh complete medium containing 5-FU, Cu_2_O@TA, or Cu_2_O@TA@5-FU at specific mass concentrations (5, 10, 20, 40, 80, and 120 μg/mL). Cytotoxicity was subsequently determined after a 24-h co-incubation period.

For cell viability evaluation, 4T1 cells were uniformly seeded into 96-well plates. After cell adhesion, the cells were randomly divided into six groups: (1) nanomaterial alone: Cu_2_O@TA; (2) free drug alone: 5-FU; (3) chemodynamic therapy group: 100 μmol·L^−1^ H_2_O_2_ + Cu_2_O@TA; (4) drug therapy group: 100 μmol·L^−1^ H_2_O_2_ + 5-FU; (5) drug-loaded nanomaterial group: Cu_2_O@TA@5-FU and (6) CDT combined with drug-loaded nanomaterial group: 100 μmol·L^−1^ H_2_O_2_ + Cu_2_O@TA@5-FU. The concentration gradients of 5-FU, Cu_2_O@TA, and Cu_2_O@TA@5-FU in each group were identical to those used in the cytotoxicity assessment described above.

After completing the 24-h incubation with the respective formulations, a CCK-8 solution (representing 10% of the total well volume) was introduced into each well. The microplates were then incubated in the dark for a further 2 h to allow for color development. Finally, the optical density (OD) of the wells was recorded at 450 nm via a microplate spectrophotometer, and the relative cell viability was computed using Equation (3):

(3) $$\mathrm{Cell}\;\mathrm{viability}\;(\%)\;=\;\frac{{(OD}_{experimental\;group\;}-\;{OD}_{blank\;group})}{\left({OD}_{control\;group\;}-{\;OD}_{blank\;group}\right)}\times 100\%$$

where *OD*_experimental group_ is the absorbance of the experimental wells (containing cells, culture medium, CCK-8 reagent, and the tested formulations); *OD*_control group_ represents the absorbance of the untreated control wells (containing cells, culture medium, and CCK-8 reagent, but no drugs); and *OD*_blank group_ denotes the background absorbance of the blank wells (containing only culture medium and CCK-8 reagent, without cells).

## Data Availability

The data presented in this study are available on request from the corresponding author.

## Supplementary Materials

The following supporting information can be downloaded at https://www.mdpi.com/article/10.3390/gels12060487/s1. Figure S1: Fitting of the in vitro 5-FU release profiles from Cu_2_O@TA@5-FU to the Ritger-Peppas (Korsmeyer–Peppas) kinetic model under pH 5.0. Figure S2: DLS measurement of Cu_2_O@TA. Table S1. Elemental content based on EDS.

## References

[1] Wu X., Li Y., Wen M., Xie Y., Zeng K., Liu Y.-N., Chen W., Zhao Y. (2024). Nanocatalysts for modulating antitumor immunity: Fabrication, mechanisms and applications. Chem. Soc. Rev..

[2] Xie W., Hao Q., Ye Z., Sha R., Wen B., Wang H., Zhang H., Jia G., Le X.C., Jiang G. (2025). Spherical nucleic acids directed cryosynthesis of manganese nanoagents for tumor imaging and therapy. Angew. Chem. Int. Ed..

[3] Du S., Ma G., Li X., Chao M., Fan L., Zhao Z., Tang R., Li J., Jing C., Liu Y. (2026). Targeting ROS-metabolism dual pathways to trigger PANoptosis by cobalt vanadium oxides biomimetic nanocakes for tumor immunotherapy. Biomaterials.

[4] Fu S., Yang R., Zhang L., Liu W., Du G., Cao Y. (2025). Biomaterials biomimetic CoO@AuPt nanozyme responsive to multiple tumor microenvironmental clues for augmenting chemodynamic therapy. Biomaterials.

[5] Jiang L., Lu Q., Gong E., Liu J., Yu H., Ren B., Yang L., He S., Huang Y. (2026). A self driving and self reporting petal like Au–Cu_2_O metalloenzyme for probing H_2_S mediated cuproptosis. ACS Nano.

[6] Zeng J., Ding C., Chen L., Yang B., Li M., Wang X., Su F., Liu C., Huang Y. (2023). Multienzyme mimicking Au@Cu_2_O with complete antioxidant capacity for reactive oxygen species scavenging. ACS Appl. Mater. Interfaces.

[7] Wang H., Ma K., Li Z., Lou S., Yang D., Zhang H., Du Y., Dai S., Gong X., Liu Z. (2026). Augmenting cuproptosis and anti-metastatic immunity in breast cancer by copper based nanoplatform for synergistic immunotherapy via lactate metabolic reprogramming and hypoxia alleviation. J. Control. Release.

[8] Li Z., Yang J., Ren B., Fan Q., Huang L., Guo S., Zhou R.L., Chen S., Feng J., Yan C. (2024). Double layered hollow mesoporous cuprous oxide nanoparticles for double drug sequential therapy of tumors. Adv. Mater..

[9] Meng X., Zhou K., Qian Y., Liu H., Wang X., Lin Y., Shi X., Tian Y., Lu Y., Chen Q. (2022). Hollow cuprous oxide@nitrogen doped carbon nanocapsules for cascade chemodynamic therapy. Small.

[10] Zhang L., Shen H., Liu T., Li B., Chen X., Wang H., He C., Liu Y., Cao G., Yu S. (2025). A PH/GSH dual responsive triple synergistic bimetallic nanocatalyst for enhanced tumor chemodynamic therapy. Small.

[11] Jin L., Zhou S., Zhang T., Cui F., Yu H., Wang J., Wu M., Wang Y., Song S., Zhang S. (2025). A multi-functional cascade nanoreactor for remodeling tumor microenvironment to realize mitochondria dysfunction via ROS/Zn^2+^ ions overload. Small.

[12] Wu X., Rong J., Cui Y., Cong J., Qu X., Hu X. (2025). Engineering nanomedicine with oxidative stress amplification and antioxidant defense disruption for enhanced cancer treatment. J. Med. Chem..

[13] Sun R., Liu R., Tian Y., Li Y., Fan B., Li S. (2025). Removing barriers to tumor ‘oxygenation’: Depleting glutathione nanozymes in cancer therapy. Int. J. Nanomed..

[14] Luo X., Hu H., Pan Z., Pei F., Qian H., Miao K., Guo S., Wang W., Feng G. (2020). Efficient and stable catalysis of hollow Cu_9_S_5_ nanospheres in the Fenton-like degradation of organic dyes. J. Hazard. Mater..

[15] Park J.Y., Lee S., Kim Y., Ryu Y.B. (2024). Antimicrobial activity of morphology controlled Cu_2_O nanoparticles: Oxidation stability under humid and thermal conditions. Materials.

[16] Zhou Y., Wang Q., Wang Q., Zhou Z., Peng X., Qu B., Zhang R. (2025). Self assembled copper chlorogenic acid nanoparticles: Inducing pyroptosis and cuproptosis to activate antitumor immunity. J. Control. Release.

[17] Zhang H., Zhang J., Liu B., Xiao J., Stuart M.A.C., Hou G., Zhang H., Liang S., Li Z., Wang Q. (2024). Natural phenolic metal framework strengthened mesona chinensis polysaccharides microgels for improved viability of probiotics to alleviate the liver injury and gut microbiota dysbiosis. Adv. Funct. Mater..

[18] Liu T., Ma M., Ali A., Liu Q., Bai R., Zhang K., Guan Y., Wang Y., Liu J., Zhou H. (2024). Self assembled copper tannic acid nanoparticles: A powerful nano bactericide by valence shift of copper. Nano Today.

[19] Ma Y., Guo H., Hui Y., Peng L., Zhuang D., Niu W., Yan Y., Hao Y., Yang W. (2025). Construction of Eu^3+^ modified covalent organic framework and fluorescence detection of L-Lysine. Colloids Surf. A Physicochem. Eng. Asp..

[20] Cheng R., Jiang L., Gao H., Liu Z., Mäkilä E., Wang S., Saiding Q., Xiang L., Tang X., Shi M. (2022). A PH-responsive cluster metal organic framework nanoparticle for enhanced tumor accumulation and antitumor effect. Adv. Mater..

[21] Ali A., Javed R., Farhangi S., Shah T., Ullah S., ul Ain N., Liu T., Guo Z., Lynch I., Raza F. (2023). Metal phenolic networks (MPNs) based PH-sensitive stimulus responsive nanosystems for drug delivery in tumor microenvironment. J. Drug Deliv. Sci. Technol..

[22] Cheng B., Li D., Li C., Zhuang Z., Wang P., Liu G. (2023). The application of biomedicine in chemodynamic therapy: From material design to improved strategies. Bioengineering.

[23] Wang J., Liu Y., Cui T., Yang H., Lin L. (2024). Current progress in the regulation of endogenous molecules for enhanced chemodynamic therapy. Chem. Sci..

[24] He J., Hu Y., Liu P., Li D., Huang Y., Liu J., Xia Z., Liu H., Li W., Shen Z. (2026). Tumor microenvironment responsive copper doped carbon dots for synergistic chemotherapy and cuproptosis in laryngeal cancer. iScience.

[25] Xia Y., Gu M., Wang J., Zhang X., Shen T., Shi X., Yuan W.E. (2024). Tumor microenvironment-activated, immunomodulatory nanosheets loaded with copper(II) and 5-FU for synergistic chemodynamic therapy and chemotherapy. J. Colloid Interface Sci..

[26] Xu C., Wang J., Wang D., Qi H., Wang L., Cheng R., Ta N., Shi J., Zhang W., Chen J. (2025). Defect engineered CuO/CeO_2_ catalysts: Enhanced low temperature CO preferential oxidation through dual promotion of CO adsorption and O_2_ activation. Mol. Catal..

[27] Ding J., Xu C., Chai H., Yao X., Hao Y., Yang Y., Sun X., Fan G., Zeng S. (2025). Unveiling the decisive role of surficial properties on CuO/CeO_2_ catalysts during CO preferential oxidation. Appl. Catal. A Gen..

[28] Ding J., Xu C., Fan G., Naren T., Wang Y., Liu Y., Gu X., Wu L., Zeng S. (2023). Engineering CeO_2_ configurations to regulate the CuO dispersion and switch pathways of preferential CO oxidation. Appl. Catal. B Environ..

[29] Xu C., Li S., Zhang Y., Li Y., Zhou J., Qin G. (2019). Synthesis of CuO_x_–CeO_2_ catalyst with high density interfaces for selective oxidation of CO in H_2_-Rich stream. Int. J. Hydrogen Energy.

[30] Ding J., Yao X., Hou K., Chai H., Xu C., Zeng S., Gu X. (2026). Lattice confinement within CeO_2_ promotes Cu ion stabilization and oxygen activation during CO preferential oxidation. Chem. Eng. J..

[31] Ding J., Xu C., Yao X., Hou K., Yang Y., He D., Chai H., Sun X., Zeng S. (2025). Interfacial anchoring ultrafine CuO_x_ nanoparticles on CeO_2_ via coordination chemistry to stabilize Cu^+^-O_v_-Ce^4+^ active sites for CO preferential oxidation. ACS Appl. Mater. Interfaces.

[32] Wang D., Xu C., Wu S., Li G., Zhang H., Qi H., Cheng R., Bao L., Guo H., Chen J. (2026). Engineering Fe_3_O_4_–Fe_3_C/C heterojunction nanosheets with multimodal therapy for tumor microenvironment-programmed drug delivery and enhanced chemodynamic therapy. Dalt. Trans..

[33] Li J., Li J., Wei J., Zhu X., Qiu S., Zhao H. (2021). Copper tannic acid coordinated meta organic nanosheets for synergistic antimicrobial and antifouling coatings. ACS Appl. Mater. Interfaces.

[34] Abdelkareem S., El-Sayed M.M.H., Yacoub N., Reda A., Butera V., Camellone M.F., Ritacco I., Shoeib T. (2025). Copper oxide nanoparticles as delivery vehicles for different Pt(Ii)-drugs: Experimental and theoretical evaluation. J. Mater. Chem. B.

[35] Gieroba B., Vivcharenko V., Kalisz G., Kazimierczak P., Mozgova O., Khalavka M., Nosach L., Pięta I.S., Nowakowski R., Przekora A. (2026). Molecular and physicochemical arrangement of chitosan ibuprofen matrices for topical drug delivery on skin: Preparation impact. RSC Adv..

[36] Hanumanthappa R., Venugopal D.M., Nethravathi P.C., Shaikh A., Siddaiah B.M., Heggannavar G.B., Patil A.A., Nanjaiah H., Suresh D., Kariduraganavar M.Y. (2023). Polyvinylpyrrolidone capped copper oxide nanoparticles anchored pramipexole attenuates the rotenone induced phenotypes in a drosophila parkinson’s disease model. ACS Omega.

[37] Liu X., Geng P., Yu N., Xie Z., Feng Y., Jiang Q., Li M., Song Y., Lian W., Chen Z. (2022). Multifunctional doxorubicin@hollow-Cu_9_S_8_ nanoplatforms for photothermally augmented chemodynamic chemo therapy. J. Colloid Interface Sci..

[38] Alvi A., Alqassim S., Khan N.A., Khatoon B., Akbar N., Kawish M., Faizi S., Shah M.R., Alharbi A.M., Alfahemi H. (2024). Antibacterial effects of quercetagetin are significantly enhanced upon conjugation with chitosan engineered copper oxide nanoparticles. BioMetals.

[39] Zhou T., Xie S., Zhou C., Chen Y., Li H., Liu P., Jiang R., Hang L., Jiang G. (2022). All in one second near infrared light responsive drug delivery system for synergistic chemo photothermal therapy. ACS Appl. Bio Mater..

[40] Chen W., Lin C., Gao Z., Huang Y., Wang X., Zhang Q., Zhang Y., Tan M., Hou Z. (2025). A tumor microenvironment responsive nanocomposite for enhanced copper retention and hypoxia reversal to promote cuproptosis in tumor treatment. Acta Biomater..

[41] Singh S., Pal K. (2023). Folic acid adorned alginate polydopamine modified paclitaxel/Zn-CuO nanocomplex for PH triggered drug release and synergistic antitumor efficacy. Int. J. Biol. Macromol..

[42] Sedky N.K., Fawzy I.M., Hassan A., Mahdy N.K., Attia R.T., Shamma S.N., Alfaifi M.Y., Elbehairi S.E., Mokhtar F.A., Fahmy S.A. (2024). Innovative microwave assisted biosynthesis of copper oxide nanoparticles loaded with platinum(Ii) based complex for halting colon cancer: Cellular, molecular, and computational investigations. RSC Adv..

[43] Singh S., Pal K. (2024). Polyphenol modified CuO nanorods capped by kappa-carrageenan for controlled paclitaxel release in furnishing targeted chemotherapy in breast carcinoma cells. Int. J. Biol. Macromol..

[44] Yang H., Chen X., Huang S., Dai H., Xiao Z., Fan J., Zhang H., Du K., Qin Y. (2026). A novel GSH depletor for simultaneous ferroptosis and cuproptosis activation in hepatocellular carcinoma. Biochem. Pharmacol..

[45] Qin J., Jiang Z. (2026). Ultrasound responsive calcium copper phosphate nanomaterials induce tumor cell death via the synergistic release of copper and calcium. Int. J. Mol. Sci..

[46] Wang Y., Xu Y., Song J., Liu X., Liu S., Yang N., Wang L., Liu Y., Zhao Y., Zhou W. (2024). Tumor cell-targeting and tumor microenvironment responsive nanoplatforms for the multimodal imaging guided photodynamic/photothermal/chemodynamic treatment of cervical cancer. Int. J. Nanomed..

