# Supplementary material for: Tannic Acid-Copper Coordination Gel-Coated Mesoporous Cuprous Oxide Nanoplatform for Synergistic 5-FU Chemotherapy and Enhanced Chemodynamic Therapy
Source: Gels. 2026 Jun 2;12(6):487. doi: 10.3390/gels12060487 (PMC13297744; doi:10.3390/gels12060487)
Supplement: Supplementary file 1 [file gels-12-00487-s001.zip › gels-4318660-supplementary.pdf]

Supporting Information

Tannic Acid-Copper Coordination Gel-Coated Mesoporous Cuprous Oxide Nanoplatform for Synergistic 5-FU Chemotherapy and Enhanced Chemodynamic Therapy

Wenyao Zhang †, Changjin Xu *,†, Jiuyang Wang, Riqing Cheng and Huiqing Guo *

College of Pharmacy, Inner Mongolia Medical University, Hohhot 010110, China; rqcheng@immu.edu.cn (R.C.)

* Correspondence: changjin.xu@immu.edu.cn (C.X.); ghq5@163.com (H.G.)

† These authors contributed equally to this work.

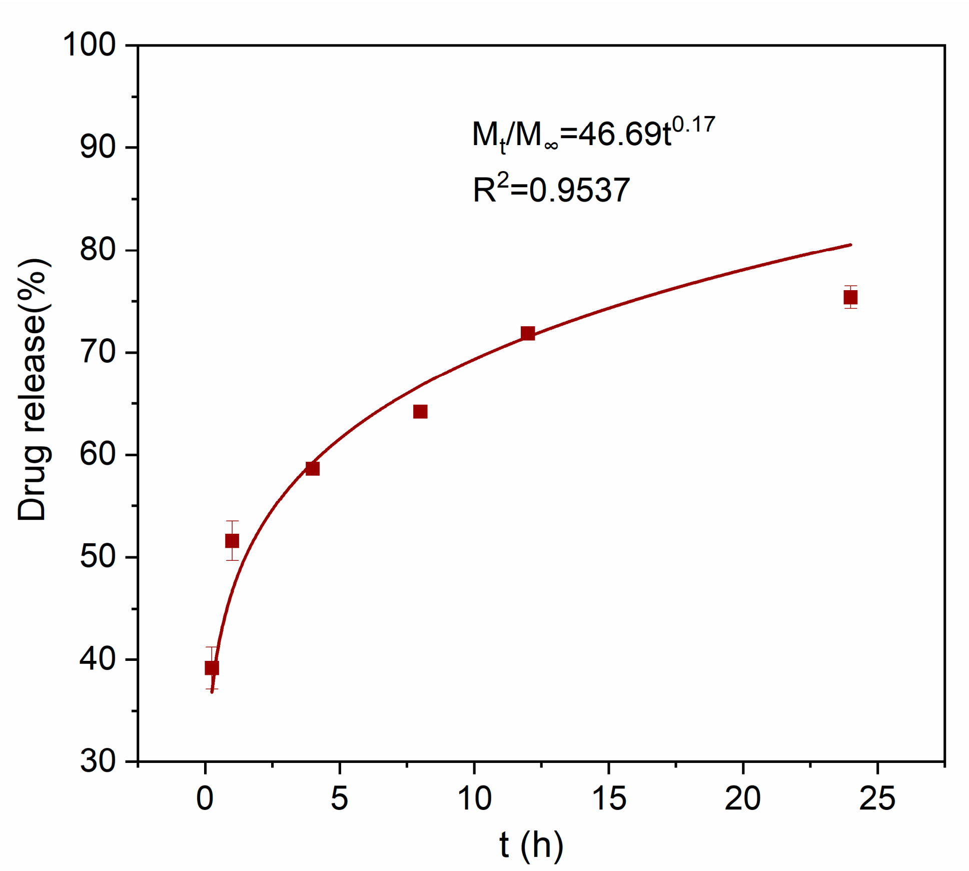

Figure S1. Fitting of the *in vitro* 5-FU release profiles from Cu_2O@TA@5-FU to the Ritger-Peppas (Korsmeyer–Peppas) kinetic model under pH 5.0.

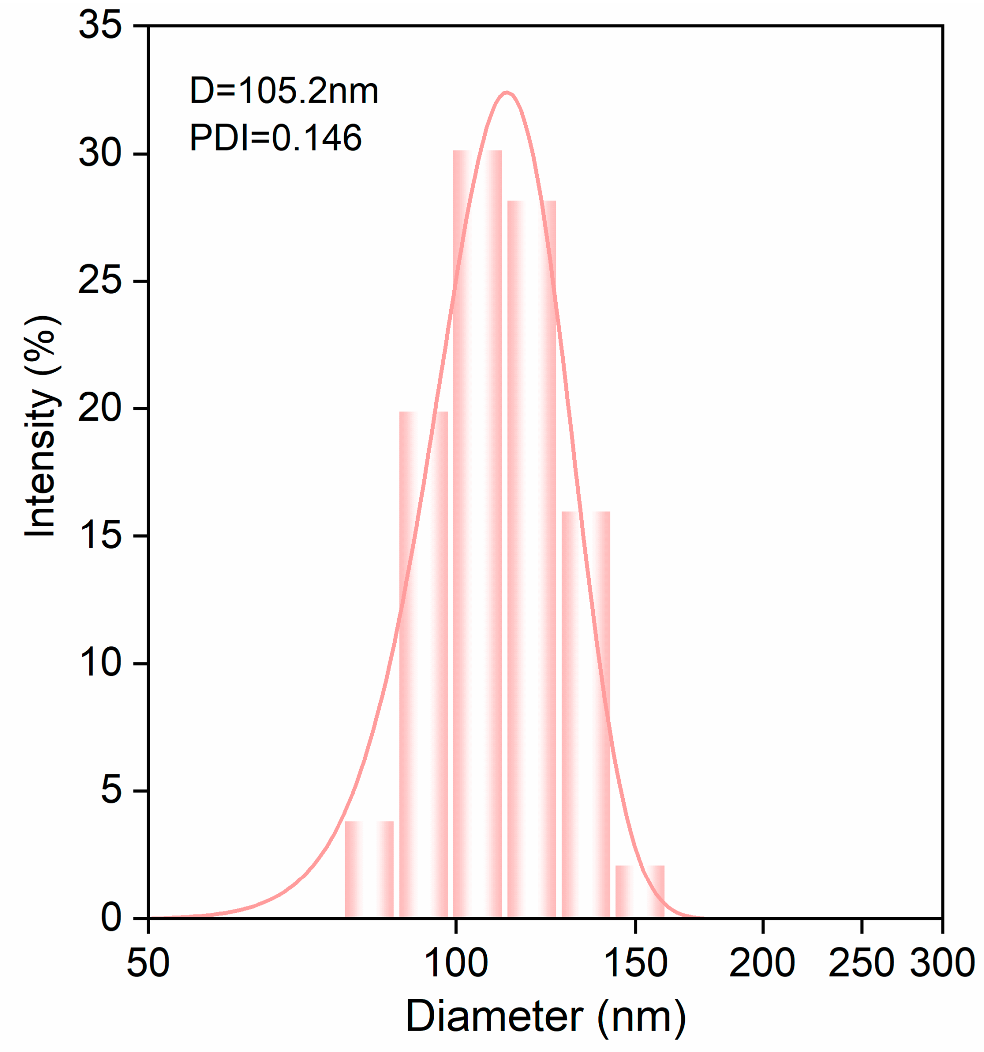

Figure S2. DLS measurement of Cu_2O@TA.

Table S1. Elemental content based on EDS

Element	Atomic Fraction (%)	Mass Fraction (%)
C	14.11	4.59
N	4.52	1.71
O	34.54	14.96
F	1.51	0.78
Cu	45.32	77.96
